# Aβ42 fibril formation from predominantly oligomeric samples suggests a link between oligomer heterogeneity and fibril polymorphism

**DOI:** 10.1098/rsos.190179

**Published:** 2019-07-10

**Authors:** Christine Xue, Joyce Tran, Hongsu Wang, Giovanna Park, Frederick Hsu, Zhefeng Guo

**Affiliations:** Department of Neurology, Brain Research Institute, Molecular Biology Institute, University of California, 710 Westwood Plaza, Los Angeles, CA 90095, USA

**Keywords:** amyloid, Alzheimer disease, oligomerization, aggregation kinetics, neurodegeneration

## Abstract

Amyloid-β (Aβ) oligomers play a central role in the pathogenesis of Alzheimer's disease. Oligomers of different sizes, morphology and structures have been reported in both *in vivo* and *in vitro* studies, but there is a general lack of understanding about where to place these oligomers in the overall process of Aβ aggregation and fibrillization. Here, we show that Aβ42 spontaneously forms oligomers with a wide range of sizes in the same sample. These Aβ42 samples contain predominantly oligomers, and they quickly form fibrils upon incubation at 37°C. When fractionated using ultrafiltration filters, the samples enriched with smaller oligomers form fibrils at a faster rate than the samples enriched with larger oligomers, with both a shorter lag time and faster fibril growth rate. This observation is independent of Aβ42 batches and hexafluoroisopropanol treatment. Furthermore, the fibrils formed by the samples enriched with larger oligomers are more readily solubilized by epigallocatechin gallate, a main catechin component of green tea. These results suggest that the fibrils formed by larger oligomers may adopt a different structure from fibrils formed by smaller oligomers, pointing to a link between oligomer heterogeneity and fibril polymorphism.

## 1. Introduction

Protein aggregation is involved in a wide range of human diseases [1]. The end aggregation product is often the pathological hallmark in these disorders. For example, amyloid-β (Aβ) aggregation leads to the formation of senile plaques in Alzheimer's disease [2]; α-synuclein forms Lewy bodies in Parkinson's disease [3]; and the tangles in various tauopathies are the result of tau aggregation [4]. The end aggregation product of these proteins is called amyloid [5], which has well-defined histochemical, biophysical and biochemical characteristics [6,7]. To understand the pathology in these amyloid-related disorders, the process of protein aggregation that leads to amyloid formation has attracted extensive research efforts. At the same time, it has been well established that protein aggregation also leads to the formation of soluble intermediates, which are often called oligomers. These oligomers are shown to be more toxic than the amyloid fibrils in a variety of activity assays using cultured cells and transgenic animals (reviewed in [8]). Some studies suggest that oligomers eventually are converted to amyloid fibrils [9,10] or form the building blocks of amyloid fibrils [11–15], and other studies show that the amyloid fibrils can also promote the formation of oligomers [16]. The relationship between oligomers and amyloid fibrils in the aggregation process is critical for a complete understanding of the underlying aggregation process and for a rational approach to develop therapeutic interventions.

In Alzheimer's disease, the senile plaques are mainly composed of the amyloid fibrils of Aβ protein [2]. Aβ is not a homogeneous species, consisting of two main isoforms: Aβ40 and Aβ42, and other minor truncated and modified Aβ variants. Aβ40 is the most abundant Aβ isoform in the brain [17–19], but Aβ42 is the major Aβ isoform in the amyloid plaques [20–23]. Experimental evidence suggests that Aβ42 and Aβ40 form interlaced amyloid fibrils [24] and they can seed each other's aggregation [25–27]. Aβ fibrillization starts with a nucleation process in which small fibril nuclei are formed [28]. These fibril nuclei adopt the structure of the fibrils and are capable of growth by monomer addition. Fibrils are also capable of promoting nucleation through a secondary nucleation process [16]. Aβ oligomers have been shown to adopt different structures from fibrils [29–31]. The relationship between oligomerization and fibrillization is not fully understood. Some studies suggest a fibrillization model of nucleated conformational conversion [9,10,30]. This model starts with the formation of Aβ oligomers, which then convert to fibril nuclei. There are also studies suggesting that Aβ forms ring-like oligomers, which form the building blocks of amyloid fibrils [15,32,33]. It is unclear how Aβ aggregation proceeds in the brain.

To this end, here we report that Aβ42 aggregation starts with oligomerization. In phosphate-buffered saline (PBS), Aβ42 forms oligomers almost immediately without fibril formation. Monomers account for only a small percentage of the Aβ42 population. Upon incubation at 37°C, the Aβ42 sample with predominantly oligomers proceeds to form fibrils and display sigmoidal aggregation kinetics. We further show that the Aβ42 sample enriched with smaller oligomers form fibrils at a faster rate than Aβ42 sample enriched with larger oligomers. Aβ42 fibrils formed by small and large oligomers appear to have different structural characteristics, suggesting a link between oligomer heterogeneity and fibril polymorphism.

## 2. Results and discussion

### 2.1. Aβ42 quickly forms oligomers with a wide range of sizes

**Figure 1.**
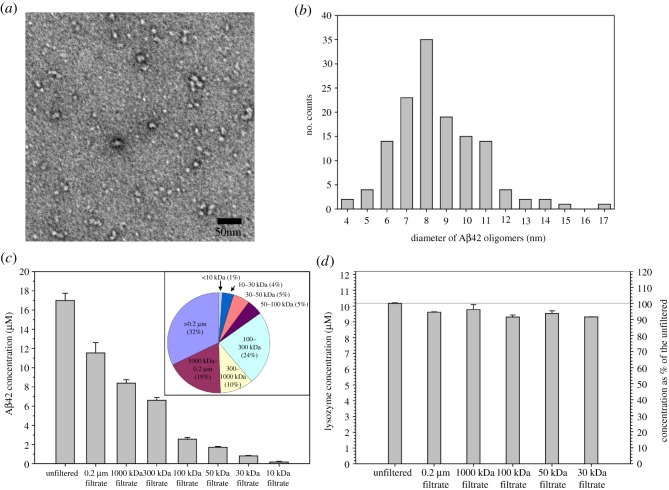
Aβ42 spontaneously forms globular oligomers of a wide range of sizes. (*a*) Transmission electron micrograph of Aβ42 samples after solubilization in a high-pH denaturing buffer and buffer exchange to PBS. (*b*) Distribution of the diameter size of Aβ42 oligomers on the electron micrograph in panel *a*. (*c*) Concentrations of Aβ42 sample upon filtration through ultrafiltration filters of various pore sizes. Error bars are standard deviations of three independent concentration measurements. Inset: percentages of different ranges of oligomer sizes. (*d*) Concentrations of hen egg white lysozyme upon filtration through ultrafiltration filters of various pore sizes. The filtration experiments were repeated three times and the error bars are standard deviations of the triplicate data. Note that different filtrates show similar concentrations for the homogeneous monomeric lysozyme.

For all the studies in this work, we used recombinant Aβ42 proteins with a protocol that we optimized over the years. The recombinant Aβ42 protein in this work does not contain any extra residues. All the purification steps were performed in high-pH buffers in the presence of chemical denaturants to limit the formation of Aβ aggregates. Following lyophilization, the Aβ42 powder was treated with hexafluoroisopropanol (HFIP). HFIP treatment has become a common practice in Aβ aggregation studies. To study aggregation, Aβ needs to be dissolved in an aqueous buffer, which is often the PBS buffer. For this purpose, we first dissolved Aβ42 protein in a high-pH denaturing buffer containing 7 M guanidine hydrochloride at pH 11. Both high pH and high concentrations of denaturant help inhibit Aβ aggregation. Then we performed a buffer exchange step with a desalting column to transfer Aβ protein to PBS buffer. We checked the morphology of the Aβ sample after buffer exchange using transmission electron microscopy (TEM), which shows that the Aβ42 sample contains abundant oligomers (figure 1*a*). These oligomers are largely globular, with diameters ranging from 4 to 15 nm (figure 1*b*). Most of the oligomers have diameters of 6–11 nm. Oligomers of similar sizes have also been previously reported for both Aβ40 and Aβ42 [13,34,35].

It was a surprise for us to see abundant oligomers in the Aβ42 sample. The time it took from solubilizing Aβ42 in the denaturing buffer to the preparation of TEM grids was approximately 1 h. The Aβ42 sample was kept on ice with the exception of the buffer exchange step, which took approximately 10 min. And the Aβ42 concentration was approximately 50 µM in the high-pH denaturing buffer before the buffer exchange step and was approximately 20 µM after buffer exchange to PBS. As a reference to the larger context of Aβ42 oligomer preparation, preparation of Aβ-derived diffusible ligands (ADDLs) requires incubation of 100 µM Aβ42 at 4°C for 24 h [36]. Aβ42 globulomers requires incubation of 100 µM Aβ42 at 37°C for 24 h [37]. Therefore, we expected that the Aβ42 sample consists of predominantly monomers. However, this unexpected outcome provides an opportunity to study the oligomer distribution and fibril formation starting from these oligomers. Previously, fast oligomerization has also been observed for both Aβ40 and Aβ42 [9,13,34,35,38,39], suggesting that oligomerization may be the first step of Aβ aggregation.

Next we studied the size distribution of Aβ42 oligomers. Our approach was to take advantage of the ultrafiltration filters that are available in a wide range of pore sizes. We took aliquots of the Aβ42 sample immediately after the buffer exchange step and filtered through ultrafiltration filters with seven molecular weight cutoffs: 10, 30, 50, 100, 300, 1000 kDa and 0.2 µm. Then we measured the concentration of the different filtrate samples using the fluorescamine method [40]. If all Aβ42 proteins are monomers, which are 4.5 kDa in size, then the filtrate concentration from various filters would be very similar. Differences in filtrate concentration, on the other hand, would reflect the distribution of Aβ42 oligomer size. Figure 1*c* shows the Aβ42 concentrations after filtering through various ultrafiltration filters. The filtrate concentrations decrease with decreasing pore sizes of the filters, consistent with the existence of large oligomers of various sizes. If we assume that most of the oligomers larger than the nominal size of the filter will stay in the retentate, and most of the oligomers smaller than the nominal size of the filter will stay in the filtrate, then the concentration difference between two filtrates would reflect the amount of oligomers whose sizes are in between the two filter sizes. For example, the concentration of the unfiltered sample in figure 1*c* is 17.0 µM, and the concentration of the 0.2 µm filtrate is 11.5 µM, therefore, the concentration of the existing Aβ42 aggregates that are larger than 0.2 µm is the concentration difference, 5.5 µM, or 32% of the total Aβ42 sample before filtration. With the same calculation, we obtained the oligomer distribution of various size groups, and this is shown as a pie chart in figure 1*c*. Although this pie chart represents a rough estimate of oligomer size distributions, it still gives us an idea of the relative population of different oligomers. We were surprised to see that the concentration of the 10 kDa filtrate is only 1% of the unfiltered sample. Even the concentration of the small oligomers in 100 kDa filtrate is only approximately 15% of the total Aβ.

As a control, we filtered hen egg white lysozyme, a monomeric protein of 14 kDa, through 30, 50, 100, 1000 kDa, and 0.2 µm filters. The results for lysozyme show that the concentrations of the filtrate are greater than 90% of the unfiltered sample for all the filters (figure 1*d*). The 50 kDa, 1000 kDa and 0.2 µm filtrates are 94–96% of the unfiltered sample, and the 30 kDa and 100 kDa filtrates are 91–92% of the unfiltered sample. The filtration studies of lysozyme provide support for the validity of the filtration method to study oligomer size distribution.

We realize that studying oligomer size distribution with ultrafiltration filters seems to be a crude method at first glance. But we argue that ultrafiltration is actually an excellent approach for the following reasons. First, although each individual filter is not capable of resolving different sizes of oligomers, combination of filters with different molecular weight cutoffs can provide a picture that is not available through other means. Second, the time required for ultrafiltration is only 10–20 min and the filtration step can be performed at low temperatures using refrigerated centrifuge. This will help limit further oligomerization and fibrillization. One could argue that size exclusion chromatography may give better separation of oligomers of different sizes. In practice, we have not seen any reports that show discreet size exclusion peaks corresponding to Aβ oligomers of different sizes. There are reports in which researchers have used fraction numbers as an indication of oligomer size. When used this way, size exclusion chromatography lacks the resolution required to distinguish Aβ oligomers of different sizes. Ultrafiltration filters have been used by other groups as a way to separate Aβ oligomers into different size groups, and have successfully shown that oligomers separated using ultrafiltration filters have different bioactivities [41,42].

**Figure 2.**
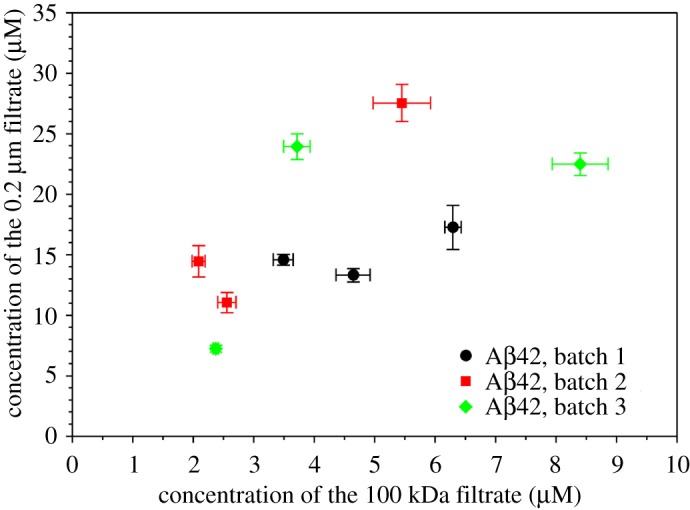
Batch variations of Aβ42 oligomer size distributions. Aβ42 samples of three batches, three samples from each batch, were filtered through 100 kDa and 0.2 µm filters, and concentrations were measured. Error bars are the standard deviations of three independent concentration measurements.

We next investigated the sample-to-sample and batch-to-batch variations in terms of the size distribution of Aβ42 oligomers. We studied three purification batches of Aβ42. Each batch of Aβ42 was prepared from independently cultured *E. coli* cells, and multiple tubes of Aβ42 were obtained from each batch of preparation. Within each batch, we investigated three tubes of Aβ42 sample. In total, nine tubes of HFIP-treated Aβ42 powder were separately dissolved in the high-pH denaturing buffer called CG. The concentration of Aβ42 in CG buffer was 50 µM for all nine tubes. After buffer exchange to PBS buffer, two aliquots of 400 µl from each sample were filtered through 0.2 µm or 100 kDa filters, and the concentration of the filtrate was measured using the fluorescamine method [40]. The results of measured concentrations are shown in figure 2. The concentrations of both the 0.2 µm and 100 kDa filtrates show a wide range even though the starting concentrations in CG buffer are all the same at 50 µM. These results suggest that the initial oligomerization process is controlled by a stochastic factor. As a result, the oligomer size distribution is not reproducible from one tube of sample to another. This stochastic control of oligomerization may be similar in principle to the stochastic nucleation for Aβ aggregation proposed by Fändrich and co-workers [43].

### 2.2. Aβ42 samples enriched with smaller oligomers form fibrils faster than Aβ42 samples enriched with larger oligomers

**Figure 3.**
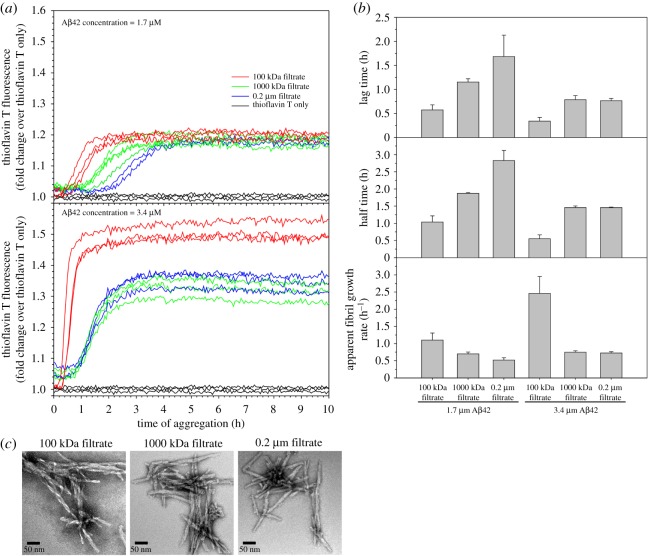
Smaller Aβ42 oligomers form fibrils faster than larger oligomers. (*a*) Fibrillization kinetics for Aβ42 samples upon filtration using three ultrafiltration filters (100 kDa, 1000 kDa, and 0.2 µm). The aggregation experiments were set up at two Aβ42 concentrations: 1.7 µM and 3.4 µM. Three repeats were performed for each filtrate and concentration. Aggregation was monitored with thioflavin T fluorescence and the same thioflavin T concentration (20 µM) was used for all the aggregations. The fluorescence data were presented using fold change, which was calculated by dividing the fluorescence reading by the average of the thioflavin T only samples at each time point of measurement. (*b*) Kinetic parameters, lag time, half time and fibril growth rate, were extracted from the aggregation data by fitting to a sigmoidal function (see Material and methods). (*c*) Morphology of aggregated Aβ42 samples as described in panel *a*. The samples were taken at 24-h time point of aggregation and examined with TEM. Note that Aβ42 oligomers of different sizes all lead to the formation of fibrils.

We then investigated how these Aβ42 samples form fibrils after ultrafiltration. We studied three filtrate samples using the 100 kDa, 1000 kDa and 0.2 µm filters. These Aβ42 filtrates all started from a single tube of HFIP-treated Aβ42, which was dissolved in the high-pH denaturing buffer, buffer exchanged to PBS, and then split into three aliquots for ultrafiltration with 100 kDa, 1000 kDa and 0.2 µm filters. The 100 kDa filtrate represents Aβ42 monomers and small oligomers; 0.2 µm filtrate has a majority of larger oligomers; and the 1000 kDa filtrate represents a sample that is devoid of largest oligomers. After concentration measurements using fluorescamine, we set up fibrillization experiments at 37°C without agitation. Two Aβ42 concentrations at 1.7 and 3.4 µM, and three repeats for each concentration, were incubated in a plate reader to allow continuous monitoring of aggregation. The aggregation curves are shown in figure 3*a*. The aggregation data were fitted to a sigmoidal equation to obtain the lag time, half time and apparent fibril growth rate (see Material and methods and electronic supplementary material, figure S1). The 100 kDa filtrate samples form fibrils faster than both the 1000 kDa and 0.2 µm filtrates, at both 1.7 µM and 3.4 µM concentrations. At lower concentration (1.7 µM), the 1000 kDa filtrate forms fibrils faster than the 0.2 µm filtrate, but the difference between the 1000 kDa and 0.2 µm filtrates disappeared at higher concentration (3.4 µM), suggesting that faster aggregation may obscure differences in aggregation kinetics between some samples.

We also noticed that the 100 kDa filtrate has faster apparent fibril growth rate than both the 1000 kDa and 0.2 µm filtrates (figure 3*b*). Furthermore, the fibril growth rate for the 100 kDa filtrate more than doubles from 1.7 to 3.4 µM Aβ42. By contrast, the 1000 kDa and 0.2 µm filtrates show marginal increase when Aβ42 concentration is increased from 1.7 to 3.4 µM. Two main factors contribute to the apparent fibril growth rate: the rate of fibril elongation and the concentration of fibril nuclei. Because the same Aβ42 protein was used, the rate of fibril elongation is likely similar for different filtrates. Therefore, we speculate that the high fibril growth rate for the 100 kDa sample is mainly due to higher concentrations of fibril nuclei than the 1000 kDa and 0.2 µm filtrates.

We examined the aggregation products after 24 h of incubation using TEM. The electron micrographs show fibrillar morphology for all three filtrate samples (figure 3*c*), confirming that the aggregation reactions we are studying here are fibrillization.

We considered the possibility that the differences in aggregation kinetics resulted from concentration differences in the aggregation reaction. In other words, we may underestimate the concentration of the 100 kDa filtrate, or overestimate the concentration of the 1000 kDa and 0.2 µm filtrates. We do not believe that this is likely for the following reasons. First, we used fluorescamine to measure the concentration of the Aβ filtrate [40]. Fluorescamine is a small molecule of 276 Da. It reacts with the primary amine groups of N-terminus or lysine. In oligomers, it is possible that the primary amines are buried inside the protein core and are thus not accessible to fluorescamine. As a result, this would lead to an underestimate of Aβ concentration. If this were the case, the concentration of larger oligomers would be underestimated compared to smaller oligomers. The aggregation kinetics show that samples with larger oligomers form fibrils slower, in contrast to this possibility. Second, the fluorescence intensity at aggregation plateau suggests that our concentration measurement is accurate. Xue *et al*. [44] showed that thioflavin T fluorescence is a quantitative measure of fibril quantity. For Aβ42 aggregation at 1.7 µM, all three filtrates reach similar fluorescence intensity at aggregation plateau. For Aβ42 aggregation at 3.4 µM, the 100 kDa filtrate reached a higher fluorescence intensity than the 1000 kDa and 0.2 µm filtrates. We speculate that the 100 kDa filtrate forms fibrils of a different structure from the 1000 kDa and 0.2 µm filtrates, and this point is further discussed below. Third, even if we compare the 100 kDa filtrate at 1.7 µM with the 1000 kDa and 0.2 µm filtrates at 3.4 µM, the 100 kDa filtrate still forms fibrils faster, and has a faster apparent fibril growth rate, suggesting that the difference in fibrillization kinetics between smaller and larger oligomers is not due to inaccurate concentration measurements.

**Figure 4.**
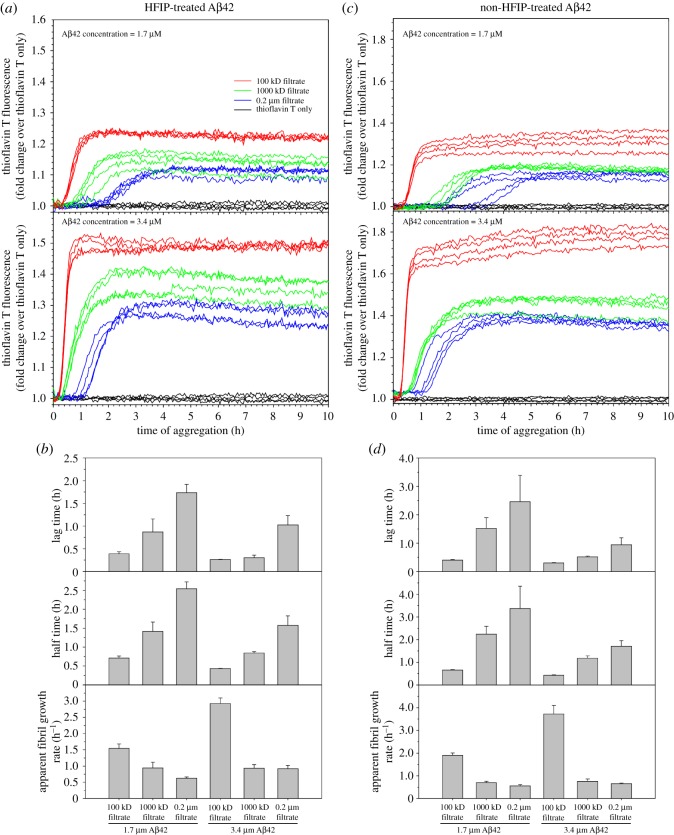
Batch variations and the effect of HFIP treatment on the fibrillization of different Aβ42 filtrates. (*a*,*b*) Aβ42 sample of a different batch from the one in figure 3 was used for the ultrafiltration and aggregation. Four repeats of each filtrate and concentration were studied. The aggregation curves are shown in *a*, and kinetic parameters from fitting to a sigmoidal function are shown in *b*. (*c*,*d*) Aβ42 sample of the same batch as the one in figure 3, but without HFIP treatment, was used for ultrafiltration and aggregation. Four repeats of each filtrate and concentration were studied. The aggregation curves are shown in *c*, and kinetic parameters from fitting to a sigmoidal function are shown in *d*.

We next investigated the reproducibility of our observation with a different Aβ preparation batch (figure 4*a,b*), and an Aβ sample without HFIP treatment (figure 4*c,d*). In both cases, we observed that the 100 kDa filtrate forms fibrils faster than the 0.2 µm filtrate, and the 1000 kDa filtrate forms fibrils at a rate that is in between the 100 kDa and 0.2 µm filtrates. Similarly, we also observed that the fibril growth rate of the 100 kDa filtrate increases dramatically from 1.7 to 3.4 µM Aβ42, while the 1000 kDa and 0.2 µm filtrates show marginally higher fibril growth rate at higher Aβ42 concentrations.

**Figure 5.**
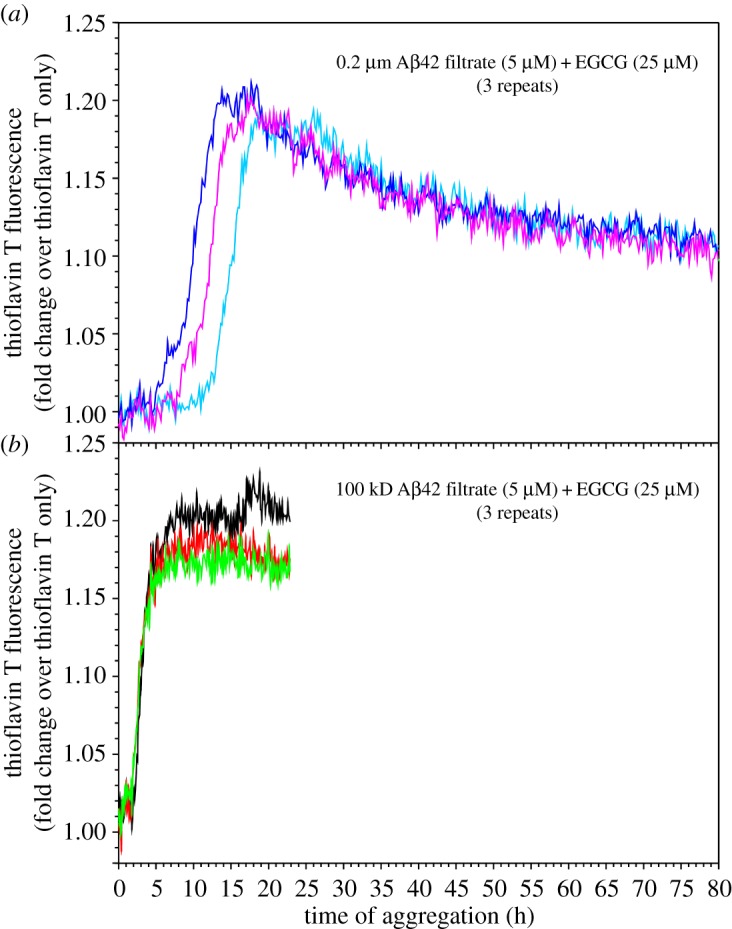
Aβ42 fibrillization in the presence of EGCG. Aggregation of Aβ42 samples (5 µM) filtered through 0.2 µm (*a*) and 100 kDa (*b*) filters was set up in the presence of EGCG (25 µM). Note that the 0.2 µm filtrate sample shows a gradual decrease in fluorescence after the fibril growth phase, which is attributed to the solubilization of Aβ42 fibrils by EGCG. By contrast, the fluorescence signal for the 100 kDa filtrate remains stable after the aggregation reaches plateau.

In addition to the faster fibrillization rate, smaller Aβ42 oligomers often show higher thioflavin T fluorescence at aggregation plateau, especially at the higher concentration that we studied (figures 3 and 4). Xue *et al*. [44] previously showed that thioflavin T fluorescence is a quantitative measure of fibril amount. Since we are comparing fibril formation at the same Aβ42 concentration, the difference in thioflavin T fluorescence suggests a possibility that the underlying fibril structure, not just the amount of fibrils, is different for smaller and larger oligomers. Although the best evidence may come from the structures of fibrils formed by the smaller and larger oligomers, structural determination of these fibrils is a challenging task in itself and is beyond the scope of this work. However, there are other ways to study the structure based on the relationship between protein structure and its thermodynamic stability. In a separate study of the Guo laboratory (unpublished data, GP & ZG, 2019), we have found that epigallocatechin gallate (EGCG), a main component of green tea catechins, is capable of solubilizing Aβ fibrils, and this effect depends on how the fibrils were prepared. Therefore, we investigated how EGCG affects the fibrillization of the 100 kDa and 0.2 µm filtrates. Figure 5 shows the aggregation of 5 µM Aβ42, filtered through 100 kDa or 0.2 µm filters, in the presence of 25 µM EGCG. For the 0.2 µm filtrate, EGCG leads to a steady decrease in thioflavin T fluorescence after the fibril growth phase (figure 5*a*). By contrast, the thioflavin T fluorescence for the 100 kDa filtrate remained stable after the aggregation has reached plateau (figure 5*b*). These results suggest a difference in stability for the fibrils formed by the 100 kDa and 0.2 µm filtrates. While the 0.2 µm fibrils can be solubilized, although not completely, by EGCG, the 100 kDa fibrils are resistant to EGCG solubilization at this combination of Aβ and EGCG concentrations. We attribute this difference to the underlying structure of the 100 kDa and 0.2 µm fibrils.

**Figure 6.**
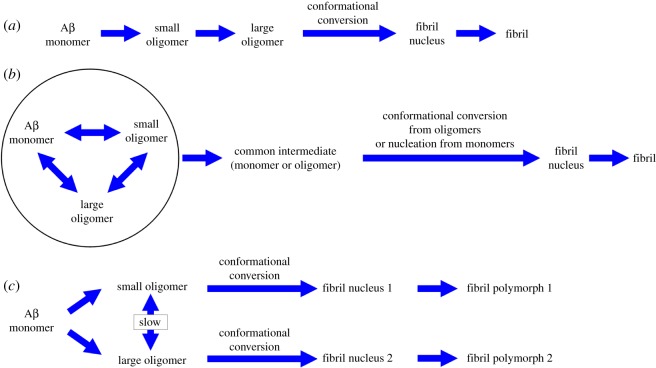
Implications on potential fibrillization pathways of Aβ. (*a*) Aβ first forms oligomers, which grow in size and eventually convert to fibrils. This model predicts that larger oligomers form fibrils faster than smaller oligomers. (*b*) Aβ monomers and different oligomers are in fast equilibrium and different Aβ species form fibrils through a common intermediate. This model predicts that Aβ from different starting states may form fibrils with different length of lag phase, but should have similar characteristics for the fibril growth phase (such as fibril growth rate and fluorescence at aggregation plateau). (*c*) Aβ aggregates to form different oligomers, and the interconversion between oligomers is slow compared to fibrillization. Different oligomers form fibrils independently, and could form fibrils of different structures.

The fibrillization kinetics of different Aβ42 oligomers have implications regarding the mechanism of Aβ aggregation. Here we consider three potential pathways of Aβ aggregation that involves Aβ oligomers. In the first pathway (figure 6*a*), Aβ aggregates to form oligomers, and then oligomers grow in size and eventually form fibrils. Our results are not in favour of this pathway because larger oligomers form fibrils at a slower rate (figures 3 and 4). In the second pathway (figure 6*b*), Aβ monomers and oligomers are in fast equilibrium, and different Aβ species form fibrils through a common step, such as nucleation from monomers. This pathway predicts that different filtrates would form fibrils with similar fibril growth rate and similar final thioflavin T fluorescence, with the main difference being lag time. Our results are not consistent with this model (figures 3 and 4). In the third pathway (figure 6*c*), Aβ aggregates to form oligomers of different sizes, and different oligomers proceed to form fibrils without interconversion between different oligomers. This would explain the different thioflavin T fluorescence amplitude from different oligomers upon fibrillization, and the potential structural difference between these fibrils (figure 5). The step of oligomer to fibril formation is via nucleated conformation conversion, which has been proposed previously for Aβ [9,10]. Similar mechanisms have also been proposed for other amyloid systems [45,46]. The contribution of our model here is to include different types of oligomers and to suggest that different oligomers form fibrils in parallel fibrillization pathways. Therefore, our model suggests that Aβ42 samples of smaller oligomers would form a higher concentration of fibril nuclei, consistent with the higher apparent fibril growth rate (figures 3 and 4). The shorter lag time for the fibrillization of smaller oligomers is likely due to a faster conversion rate from oligomers to fibril nuclei. Previously, we have also suggested that the mechanism of this nucleated conformational conversion is a β-strand rotation, which converts antiparallel β-structure in oligomers to parallel β-sheet structure in fibril nuclei [30]. A similar structural conversion was also proposed by Fu *et al*. [10] based on NMR studies.

Our model of Aβ aggregation suggests a link between oligomer heterogeneity and fibril polymorphism. The issue of fibril polymorphism has been long recognized in the amyloid research community [47]. Different fibril polymorphs have been observed even in the same sample prepared either *in vitro* [48] or *in vivo* [49], and may lead to different pathology in Alzheimer's disease. Different underlying structures of Aβ fibrils have been found in different clinical subtypes of Alzheimer's disease patients [50–52]. Our results suggest that Aβ42 oligomers of different sizes may form fibrils of different structures. Therefore, oligomer heterogeneity leads to fibril polymorphism. The heterogeneity of oligomers is likely a result of the stochastic nature of the oligomerization process. Due to the presence of two hydrophobic regions (the central hydrophobic cluster and the C-terminal region) in Aβ sequence and their importance in Aβ aggregation, the initial oligomerization process is likely driven by hydrophobic collapse. Similar to protein folding where the transition state is similar to the native state of a folded protein [53], the structures of the oligomers also dictate the final structures of fibrils that they eventually form. Given the diversity of Aβ42 oligomers that have been identified *in vitro* and *in vivo*, and the highly polymorphic nature of Aβ42 fibrils, our results here point to the potential convergence of these two lines of research inquires.

## 3. Material and methods

### 3.1. Preparation of Aβ42 proteins

Detailed protocols for Aβ42 preparation have been described previously [25,44]. Briefly, Aβ protein was expressed in *Escherichia coli* as a fusion protein, GroES-ubiquitin-Aβ, and was purified using a nickel column in a high-pH denaturing buffer. After purification, the fusion protein was cleaved to obtain full-length Aβ. Full-length Aβ was then buffer exchanged to 30 mM ammonium acetate (pH 10). At this stage, Aβ42 proteins in ammonium acetate from several purifications were pooled together, and then were aliquoted to small volumes in 1.5-ml microcentrifuge tubes for lyophilization. This pooled Aβ42 sample is called a purification batch. Each batch of Aβ consists of multiple tubes of Aβ powder, and each tube of Aβ is identical to other tubes within the same batch. The lyophilized Aβ powder was then treated with HFIP by incubating at approximately 100 µM Aβ concentration at room temperature with shaking (1000 r.p.m.) for 24 h. The HFIP was allowed to dry overnight in a chemical hood. The resulting Aβ film is stored at −80°C.

### 3.2. Ultrafiltration to prepare various Aβ42 filtrate samples

HFIP-treated Aβ42 was dissolved in a high-pH denaturing buffer called CG (20 mM CAPS, 7 M GdnHCl, pH 11) to a final Aβ concentration of 30–50 µM. Then the Aβ sample was buffer exchanged to PBS using a 5-ml HiTrap desalting column (GE Healthcare). Depending on the requirement of Aβ volume, multiple tubes of Aβ may be prepared and pooled together after buffer exchange step. Following buffer exchange, 400 µl of Aβ was pipetted to ultrafiltration filters of different molecular weight cutoff sizes ranging from 10 kDa to 0.2 µm and centrifuged at 4°C at 14 000*g*. For all the experiments described here, the ultrafiltration filters of 10, 30, 50, 100, 300 and 1000 kDa were obtained from Sartorious (Vivaspin 500 product line), and the 0.2 µm filters were obtained from PALL (Nanosep product line). All filters were pre-washed with PBS buffer. The centrifugation was monitored so that most of the sample is in the filtrate and the retentate volume is minimal.

### 3.3. Measurement of Aβ concentration using the fluorescamine method

Aβ concentration was measured using the fluorescamine method as previously described [40]. A standard curve using hen egg white lysozyme (Fisher Bioreagents, crystalline powder) was prepared for each experiment of concentration measurement. The range of amine concentrations for the standard curve is 0.3–7 µM. The volume of each assay was 50 µl, containing 500 µM fluorescamine. The assay for Aβ was performed the same way as the lysozyme standard. The Aβ sample was also diluted when needed so that the fluorescence reading is in the middle of the concentration range of the standard curve. The concentration of each Aβ sample was measured in triplicates.

### 3.4. Ultrafiltration of hen egg white lysozyme

As a control experiment, we prepared a lysozyme solution at 10.17 µM. Then 400 µl of aliquots were filtered through filters of five molecular weight cutoff sizes: 30 kDa, 50 kDa, 100 kDa, 1000 kDa and 0.2 µm. These filters were obtained from the same manufacturers as described above for Aβ ultrafiltration experiments. Following centrifugation, the lysozyme concentration of each filtrate was measured using absorbance at 280 nm and an extinction coefficient of 38 mM^−1^ cm^−1^ [54]. This ultrafiltration experiment was repeated for two additional times to obtain final concentration data in triplicates.

### 3.5. Transmission electron microscopy

Aβ samples were applied on to glow-discharged copper grids (400 mesh formvar/carbon film, Ted Pella) and stained with 2% uranyl acetate. The EM grids were then examined using a FEI T12 electron microscope with an accelerating voltage of 120 kV.

### 3.6. Fibrillization of Aβ42 samples following ultrafiltration

For each of the three aggregation experiments reported in figures 3 and 4*a,b*, one tube of Aβ42 (HFIP-treated for figures 3 and 4*a*, non-HFIP-treated for figure 4*b*) was dissolved in 1 ml of CG buffer, and then buffer exchanged to PBS. Following buffer exchange, three aliquots of 400 µl Aβ42 were filtered through the 0.2 µm, 1000 kDa and 100 kDa ultrafiltration filters at the same time using a refrigerated centrifuge. Centrifugation was monitored so that most of the volume is in the filtrate and the retentate volume is minimal. Then the concentration of the filtrates was determined using the fluorescamine method as described above. Aβ42 fibrillization assays were set up at two Aβ42 concentrations: 1.7 and 3.4 µM. A 50-µl assay was prepared by mixing 5 µl of thioflavin T stock (200 µM in PBS, freshly prepared), Aβ42 filtrate samples and PBS buffer. The thioflavin T control was prepared using 5 µl of thioflavin T stock and 45 µl of PBS buffer. The volumes of the Aβ42 filtrate and PBS were calculated based on the final Aβ42 concentration. Each aggregation condition (different filters and different Aβ42 concentrations) was performed either in triplicates or quadruplicates as indicated in the figure legends. All the mixing steps were performed on ice. Finally, the fibrillization mixtures (50 µl each) were transferred to a black 384-well Non-binding Surface microplate with clear bottom (Corning product 3655) and sealed with a polyester-based sealing film (Corning product PCR-SP). The fibrillization was started by incubation at 37°C in a Victor 3 V plate reader (Perkin Elmer) without agitation. The thioflavin T fluorescence was measured from the bottom of the plate with an excitation filter of 450 nm and an emission filter of 490 nm. The aggregation data are reported as fold change in fluorescence by dividing the average of thioflavin T fluorescence at each time point of measurements.

### 3.7. Fibrillization of Aβ42 samples in the presence of EGCG

One tube of HFIP-treated Aβ42 was dissolved in CG buffer and buffer exchanged to PBS. Then aliquots of 400 µl samples were filtered through the 0.2 µm and 100 kDa ultrafiltration filters. The concentration of Aβ filtrates was determined using the fluorescamine method described above. EGCG was obtained from Tocris (greater than 98% purity, catalogue number 4524) and was dissolved in deionized water. The concentration of EGCG was calculated based on the amount (weight) and water volume. The final aggregation was set up to have 5 µM of Aβ42 (either 0.2 µm filtrate or 100 kDa filtrate), 25 µM EGCG and 20 µM thioflavin T. All the mixing steps were performed on ice, and the aggregation was monitored using a plate reader as described above.

### 3.8. Fitting of fibrillization data

To obtain the kinetic parameters, the fibrillization data were fitted to the equation below, which was based on the sigmoidal equation described by Nielsen *et al*. [55] with modifications.

3.1 $$S=S_{I}+k_{I}\cdot t+\frac{\left(S_{F}+k_{F}\cdot t)-(S_{I}+k_{I}\cdot t\right)}{1+e^{-((t-t_{50})/\tau )}},$$

where *S* is the fluorescence intensity, *S*_I_ and *k*_I_ are the fluorescence and slope in the lag phase, *S*_F_ and *k*_F_ are the fluorescence and slope in the plateau phase, *t* is the time of aggregation, *t*_50_ is the time to reach 50% of maximal fluorescence, and *τ* is the time it takes for the fluorescence signal to change from 26.9 to 50%, or from 50 to 73.1%, of the maximum fluorescence. The lag time of aggregation is given by *t*_50_−2*τ*, and the maximum fibril growth rate is given by 1/4*τ*. The fits are shown in electronic supplementary material, figures S1–S3.

## Supplementary Material

Supplementary Figures

Reviewer comments

## Data accessibility

Data available from the Dryad Digital Repository: https://doi.org/10.5061/dryad.7n4c218 [56].

## References

[1] Chiti F, Dobson CM 2017 Protein misfolding, amyloid formation, and human disease: a summary of progress over the last decade. Annu. Rev. Biochem. 86, 27–68. (10.1146/annurev-biochem-061516-045115) 28498720

[2] Selkoe DJ 2011 Alzheimer's Disease. Cold Spring Harb. Perspect. Biol. 3, a004457 (10.1101/cshperspect.a004457) 21576255 PMC3119915

[3] Kalia LV, Lang AE 2015 Parkinson's disease. Lancet 386, 896–912. (10.1016/S0140-6736(14)61393-3) 25904081

[4] Goedert M, Eisenberg DS, Crowther RA 2017 Propagation of Tau aggregates and neurodegeneration. Annu. Rev. Neurosci. 40, 189–210. (10.1146/annurev-neuro-072116-031153) 28772101

[5] Benson MD, Buxbaum JN, Eisenberg DS, Merlini G, Saraiva MJM, Sekijima Y, Sipe JD, Westermark P 2019 Amyloid nomenclature 2018: recommendations by the International Society of Amyloidosis (ISA) nomenclature committee. Amyloid 25, 215–219. (10.1080/13506129.2018.1549825) 30614283

[6] Eisenberg DS, Sawaya MR 2017 Structural studies of amyloid proteins at the molecular level. Annu. Rev. Biochem. 86, 69–95. (10.1146/annurev-biochem-061516-045104) 28125289

[7] Iadanza MG, Jackson MP, Hewitt EW, Ranson NA, Radford SE 2018 A new era for understanding amyloid structures and disease. Nat. Rev. Mol. Cell Biol. 19, 755–773. (10.1038/s41580-018-0060-8) 30237470 PMC7617691

[8] Cline EN, Bicca MA, Viola KL, Klein WL 2018 The amyloid-β oligomer hypothesis: beginning of the third decade. J. Alzheimers Dis. 64, S567–S610. (10.3233/JAD-179941) 29843241 PMC6004937

[9] Lee J, Culyba EK, Powers ET, Kelly JW 2011 Amyloid-β forms fibrils by nucleated conformational conversion of oligomers. Nat. Chem. Biol. 7, 602–609. (10.1038/nchembio.624) 21804535 PMC3158298

[10] Fu Z, Aucoin D, Davis J, Van Nostrand WE, Smith SO. 2015 Mechanism of nucleated conformational conversion of Aβ42. Biochemistry 54, 4197–4207. (10.1021/acs.biochem.5b00467) 26069943

[11] Selivanova OM et al. 2016 Structural model of amyloid fibrils for amyloidogenic peptide from Bgl2p-glucantransferase of *S. cerevisiae* cell wall and its modifying analog. New morphology of amyloid fibrils. Biochim. Biophys. Acta 1864, 1489–1499. (10.1016/j.bbapap.2016.08.002) 27500912

[12] Selivanova OM, Suvorina MY, Surin AK, Dovidchenko NV, Galzitskaya OV 2017 Insulin and lispro insulin: what is common and different in their behavior? Curr. Protein Pept. Sci. 18, 57–64. (10.2174/1389203717666160526122421) 27226198

[13] Galzitskaya OV, Selivanova OM 2017 Rosetta stone for amyloid fibrils: the key role of ring-like oligomers in amyloidogenesis. J. Alzheimers Dis. 59, 785–795. (10.3233/JAD-170230) 28671122

[14] Selivanova OM, Surin AK, Ryzhykau YL, Glyakina AV, Suvorina MY, Kuklin AI, Rogachevsky VV, Galzitskaya OV 2018 To be fibrils or to be nanofilms? Oligomers are building blocks for fibril and nanofilm formation of fragments of Aβ peptide. Langmuir 34, 2332–2343. (10.1021/acs.langmuir.7b03393) 29338255

[15] Galzitskaya OV, Surin AK, Glyakina AV, Rogachevsky VV, Selivanova OM 2018 Should the treatment of amyloidosis be personified? Molecular mechanism of amyloid formation by aβ peptide and its fragments. J. Alzheimers Dis. Rep. 2, 181–199. (10.3233/ADR-180063) 30480261 PMC6218156

[16] Cohen SIA et al*.* 2013 Proliferation of amyloid-β42 aggregates occurs through a secondary nucleation mechanism. Proc. Natl Acad. Sci. USA 110, 9758–9763. (10.1073/pnas.1218402110) 23703910 PMC3683769

[17] Huang Y et al 2012 Effects of age and amyloid deposition on Aβ dynamics in the human central nervous system. Arch. Neurol. 69, 51–58. (10.1001/archneurol.2011.235) 21911660 PMC3254706

[18] Pannee J et al*.* 2013 A selected reaction monitoring (SRM)-based method for absolute quantification of Aβ38, Aβ40, and Aβ42 in cerebrospinal fluid of Alzheimer's disease patients and healthy controls. J. Alzheimers Dis. 33, 1021–1032. (10.3233/JAD-2012-121471) 23076076

[19] Kakuda N et al*.* 2012 Altered γ-secretase activity in mild cognitive impairment and Alzheimer's disease. EMBO Mol. Med. 4, 344–352. (10.1002/emmm.201200214) 22354516 PMC3376855

[20] Gravina SA, Ho L, Eckman CB, Long KE, Otvos L, Younkin LH, Suzuki N, Younkin SG 1995 Amyloid β protein (Aβ) in Alzheimer's disease brain. Biochemical and immunocytochemical analysis with antibodies specific for forms ending at Aβ40 or Aβ42(43). J. Biol. Chem. 270, 7013–7016. (10.1074/jbc.270.13.7013) 7706234

[21] Iwatsubo T, Odaka A, Suzuki N, Mizusawa H, Nukina N, Ihara Y 1994 Visualization of Aβ42(43) and Aβ40 in senile plaques with end-specific Aβ monoclonals: evidence that an initially deposited species is Aβ42(43). Neuron 13, 45–53. (10.1016/0896-6273(94)90458-8) 8043280

[22] Mak K, Yang F, Vinters HV, Frautschy SA, Cole GM 1994 Polyclonals to β-amyloid(1–42) identify most plaque and vascular deposits in Alzheimer cortex, but not striatum. Brain Res. 667, 138–142. (10.1016/0006-8993(94)91725-6) 7895077

[23] Miller DL, Papayannopoulos IA, Styles J, Bobin SA, Lin YY, Biemann K, Iqbal K 1993 Peptide compositions of the cerebrovascular and senile plaque core amyloid deposits of Alzheimer's disease. Arch. Biochem. Biophys. 301, 41–52. (10.1006/abbi.1993.1112) 8442665

[24] Gu L, Guo Z 2013 Alzheimer's Aβ42 and Aβ40 peptides form interlaced amyloid fibrils. J. Neurochem. 126, 305–311. (10.1111/jnc.12202) 23406382 PMC3716832

[25] Tran J, Chang D, Hsu F, Wang H, Guo Z 2017 Cross-seeding between Aβ40 and Aβ42 in Alzheimer's disease. FEBS Lett. 591, 177–185. (10.1002/1873-3468.12526) 27981583 PMC5235972

[26] Jan A, Gokce O, Luthi-Carter R, Lashuel HA 2008 The ratio of monomeric to aggregated forms of Aβ40 and Aβ42 is an important determinant of amyloid-β aggregation, fibrillogenesis, and toxicity. J. Biol. Chem. 283, 28 176–28 189. (10.1074/jbc.M803159200) PMC2661389 18694930

[27] Ono K, Takahashi R, Ikeda T, Yamada M 2012 Cross-seeding effects of amyloid β-protein and α-synuclein. J. Neurochem. 122, 883–890. (10.1111/j.1471-4159.2012.07847.x) 22734715

[28] Wetzel R 2006 Kinetics and thermodynamics of amyloid fibril assembly. Acc. Chem. Res. 39, 671–679. (10.1021/ar050069h) 16981684

[29] Gu L, Liu C, Guo Z 2013 Structural insights into Aβ42 oligomers using site-directed spin labeling. J. Biol. Chem. 288, 18 673–18 683. (10.1074/jbc.M113.457739) PMC3696641 23687299

[30] Gu L, Liu C, Stroud JC, Ngo S, Jiang L, Guo Z 2014 Antiparallel triple-strand architecture for prefibrillar Aβ42 oligomers. J. Biol. Chem. 289, 27 300–27 313. (10.1074/jbc.M114.569004) PMC4175361 25118290

[31] Huang D, Zimmerman MI, Martin PK, Nix AJ, Rosenberry TL, Paravastu AK 2015 Antiparallel β-sheet structure within the C-terminal region of 42-residue Alzheimer's β-amyloid peptides when they form 150 kDa oligomers. J. Mol. Biol. 427, 2319–2328. (10.1016/j.jmb.2015.04.004) 25889972 PMC4457621

[32] Dean DN, Das PK, Rana P, Burg F, Levites Y, Morgan SE, Ghosh P, Rangachari V 2017 Strain-specific fibril propagation by an Aβ dodecamer. Sci. Rep. 7, 40787 (10.1038/srep40787) 28098204 PMC5241678

[33] Galzitskaya O 2019 New mechanism of amyloid fibril formation. Curr. Protein Pept. Sci. 20, 630–640. (10.2174/1389203720666190125160937) 30686252

[34] Dovidchenko NV et al*.* 2016 One of the possible mechanisms of amyloid fibrils formation based on the sizes of primary and secondary folding nuclei of Aβ40 and Aβ42. J. Struct. Biol. 194, 404–414. (10.1016/j.jsb.2016.03.020) 27016282

[35] Fu Z, Aucoin DS, Ahmed M, Ziliox M, Van Nostrand WE, Smith SO. 2014 Capping of Aβ42 oligomers by small molecule inhibitors. Biochemistry 53, 7893–7903. (10.1021/bi500910b) 25422864 PMC4278677

[36] Chromy BA et al*.* 2003 Self-assembly of Aβ1-42 into globular neurotoxins. Biochemistry 42, 12 749–12 760. (10.1021/bi030029q) 14596589

[37] Barghorn S et al*.* 2005 Globular amyloid β-peptide1−42 oligomer−a homogenous and stable neuropathological protein in Alzheimer's disease. J. Neurochem. 95, 834–847. (10.1111/j.1471-4159.2005.03407.x) 16135089

[38] Elbassal EA, Morris C, Kent TW, Lantz R, Ojha B, Wojcikiewicz EP, Du D. 2017 Gold nanoparticles as a probe for amyloid-β oligomer and amyloid formation. J. Phys. Chem. C 121, 20 007–20 015. (10.1021/acs.jpcc.7b05169) PMC5737797 29276551

[39] Garai K, Frieden C 2013 Quantitative analysis of the time course of Aβ oligomerization and subsequent growth steps using tetramethylrhodamine-labeled Aβ. Proc. Natl Acad. Sci. USA 110, 3321–3326. (10.1073/pnas.1222478110) 23401512 PMC3587211

[40] Xue C, Lee YK, Tran J, Chang D, Guo Z 2017 A mix-and-click method to measure amyloid-β concentration with sub-micromolar sensitivity. R. Soc. open sci. 4, 170325 (10.1098/rsos.170325) 28878984 PMC5579099

[41] Velasco PT et al*.* 2012 Synapse-binding subpopulations of Aβ oligomers sensitive to peptide assembly blockers and scFv antibodies. ACS Chem. Neurosci. 3, 972–981. (10.1021/cn300122k) 23173076 PMC3503444

[42] Lacor PN et al*.* 2004 Synaptic targeting by Alzheimer's-related amyloid beta oligomers. J. Neurosci. 24, 10 191–10 200. (10.1523/JNEUROSCI.3432-04.2004) PMC6730194 15537891

[43] Hortschansky P, Schroeckh V, Christopeit T, Zandomeneghi G, Fändrich M 2005 The aggregation kinetics of Alzheimer's β-amyloid peptide is controlled by stochastic nucleation. Protein Sci. 14, 1753–1759. (10.1110/ps.041266605) 15987892 PMC2279324

[44] Xue C, Lin TY, Chang D, Guo Z 2017 Thioflavin T as an amyloid dye: fibril quantification, optimal concentration and effect on aggregation. R. Soc. open sci. 4, 160696 (10.1098/rsos.160696) 28280572 PMC5319338

[45] Serio TR, Cashikar AG, Kowal AS, Sawicki GJ, Moslehi JJ, Serpell L, Arnsdorf MF, Lindquist SL 2000 Nucleated conformational conversion and the replication of conformational information by a prion determinant. Science 289, 1317–1321. (10.1126/science.289.5483.1317) 10958771

[46] Nguyen HD, Hall CK 2005 Kinetics of fibril formation by polyalanine peptides. J. Biol. Chem. 280, 9074–9082. (10.1074/jbc.M407338200) 15591317

[47] Eisenberg D, Jucker M 2012 The amyloid state of proteins in human diseases. Cell 148, 1188–1203. (10.1016/j.cell.2012.02.022) 22424229 PMC3353745

[48] Meinhardt J, Sachse C, Hortschansky P, Grigorieff N, Fändrich M 2009 Aβ(1-40) fibril polymorphism implies diverse interaction patterns in amyloid fibrils. J. Mol. Biol. 386, 869–877. (10.1016/j.jmb.2008.11.005) 19038266 PMC6760659

[49] Annamalai K et al*.* 2016 Polymorphism of amyloid fibrils in vivo. Angew. Chem. Int. Ed. Engl. 55, 4822–4825. (10.1002/anie.201511524) 26954430 PMC4864496

[50] Lu J-X, Qiang W, Yau W-M, Schwieters CD, Meredith SC, Tycko R 2013 Molecular structure of β-amyloid fibrils in Alzheimer's disease brain tissue. Cell 154, 1257–1268. (10.1016/j.cell.2013.08.035) 24034249 PMC3814033

[51] Qiang W, Yau W-M, Lu J-X, Collinge J, Tycko R 2017 Structural variation in amyloid-β fibrils from Alzheimer's disease clinical subtypes. Nature 541, 217–221. (10.1038/nature20814) 28052060 PMC5233555

[52] Rasmussen J et al*.* 2017 Amyloid polymorphisms constitute distinct clouds of conformational variants in different etiological subtypes of Alzheimer's disease. Proc. Natl Acad. Sci. USA 114, 13 018–13 023. (10.1073/pnas.1713215114) 29158413 PMC5724274

[53] Daggett V, Fersht AR 2003 Is there a unifying mechanism for protein folding? Trends Biochem. Sci. 28, 18–25. (10.1016/S0968-0004(02)00012-9) 12517448

[54] Gill SC, von Hippel PH. 1989 Calculation of protein extinction coefficients from amino acid sequence data. Anal. Biochem. 182, 319–326. (10.1016/0003-2697(89)90602-7) 2610349

[55] Nielsen L, Khurana R, Coats A, Frokjaer S, Brange J, Vyas S, Uversky VN, Fink AL 2001 Effect of environmental factors on the kinetics of insulin fibril formation: elucidation of the molecular mechanism. Biochemistry 40, 6036–6046. (10.1021/bi002555c) 11352739

[56] Xue C, Tran J, Wang H, Park G, Hsu F, Guo Z 2019 Data from: Aβ42 fibril formation from predominantly oligomeric samples suggests a link between oligomer heterogeneity and fibril polymorphism *Dryad Digital Repository*. (10.5061/dryad.7n4c218) PMC6689619 31417723

