# Supplementary material for: Aβ42 fibril formation from predominantly oligomeric samples suggests a link between oligomer heterogeneity and fibril polymorphism
Source: R Soc Open Sci. 2019 Jul 10;6(7):190179. doi: 10.1098/rsos.190179 (PMC6689619; doi:10.1098/rsos.190179)
Supplement: Supplementary Figures [file rsos190179supp1.pdf]

SUPPLEMENTARY FIGURES

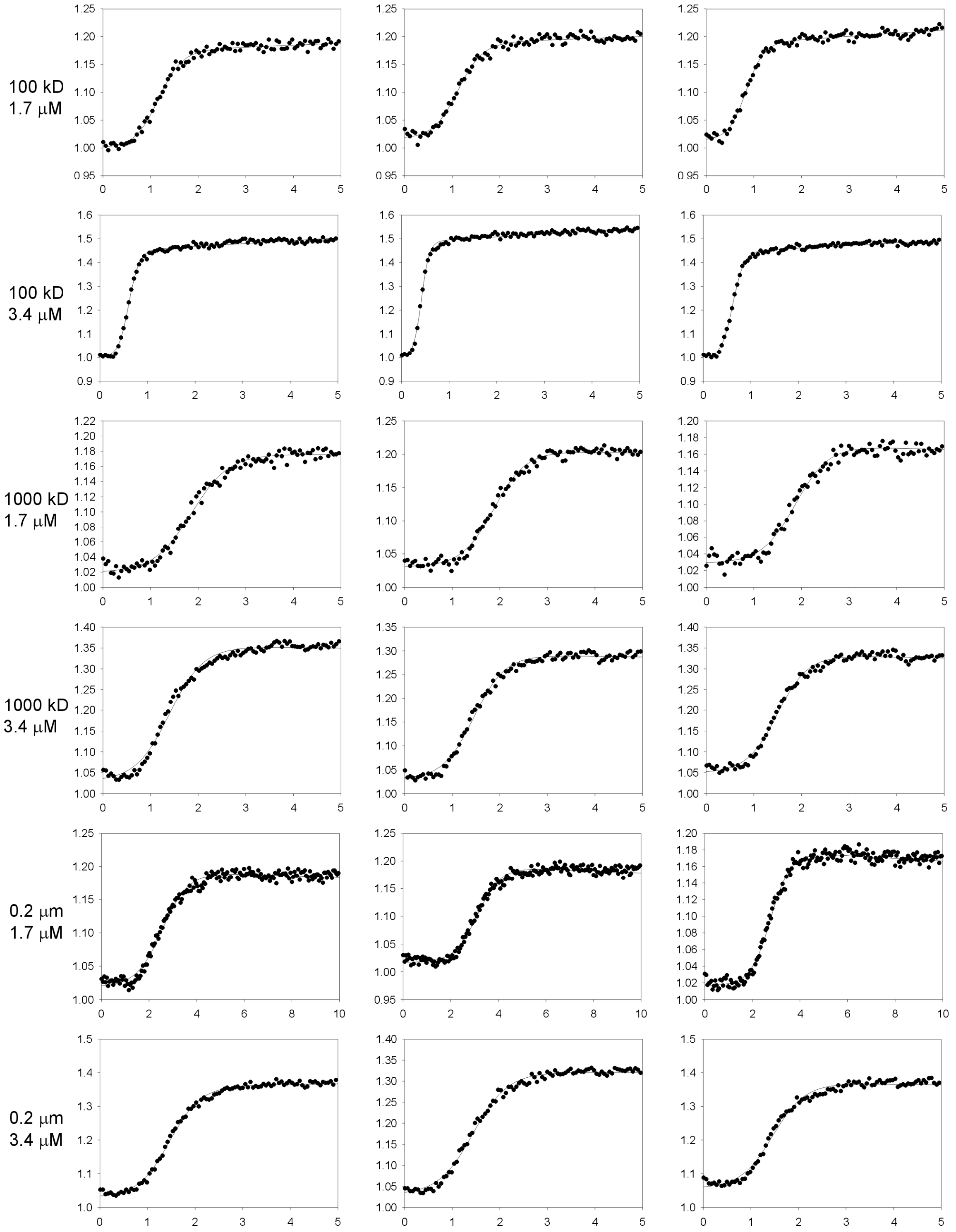

Figure S1. Fitting to a sigmoidal equation for the aggregation data presented in Figure 3a. Solid lines are best fits and the symbols are data points.

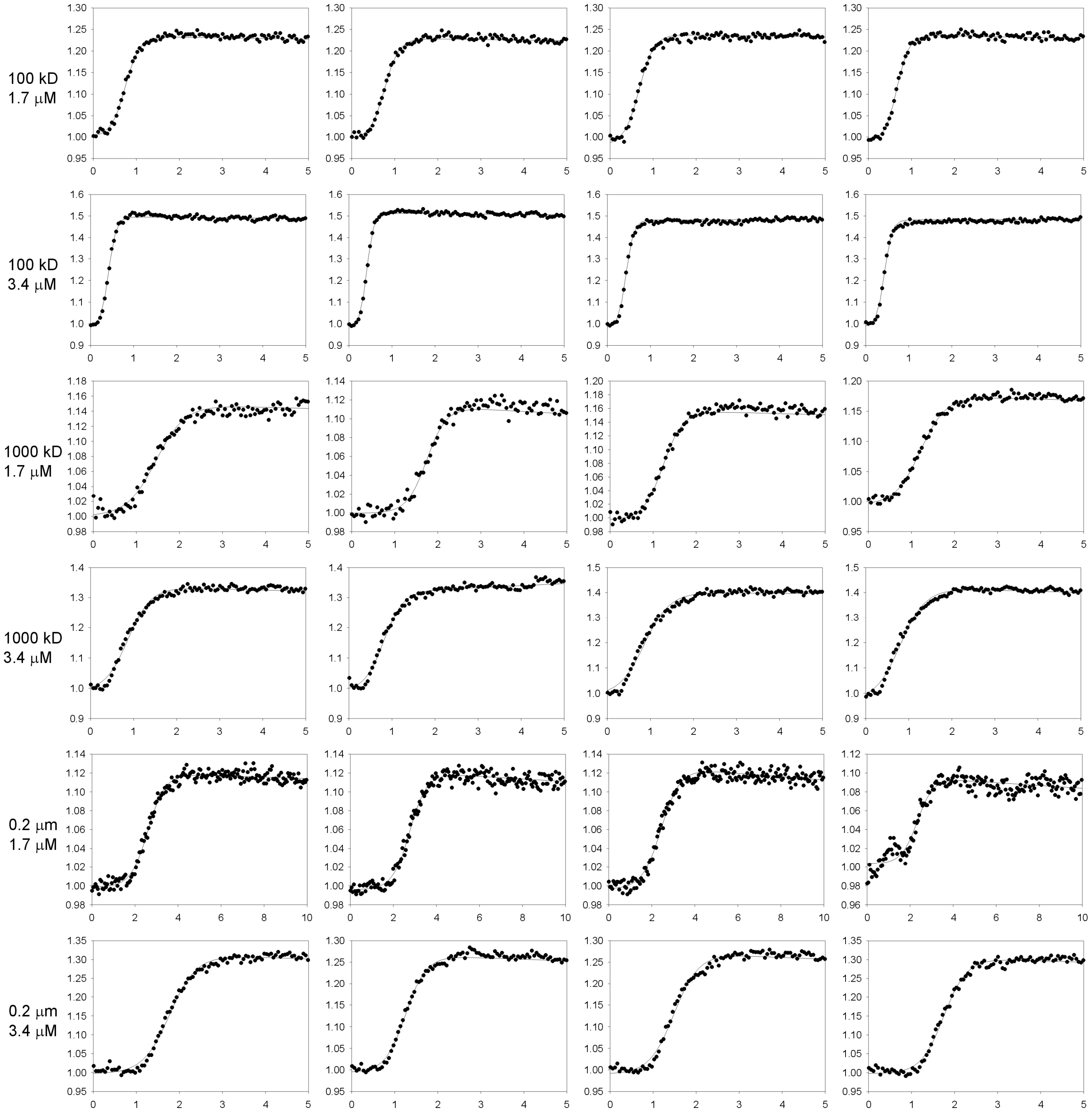

Figure S2. Fitting to a sigmoidal equation for the aggregation data presented in Figure 4a. Solid lines are best fits and the symbols are data points.

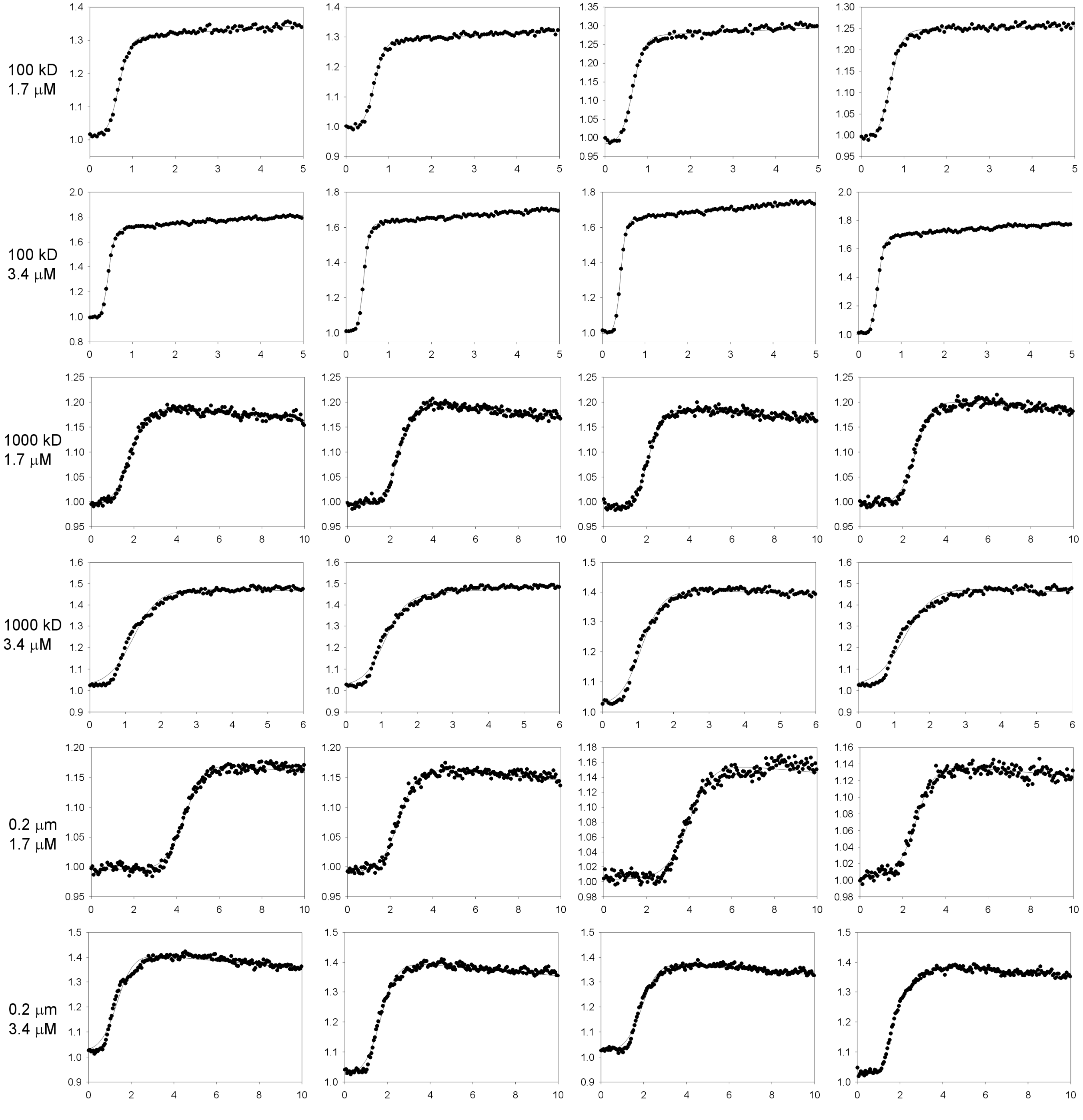

Figure S3. Fitting to a sigmoidal equation for the aggregation data presented in Figure 4c. Solid lines are best fits and the symbols are data points.
